# Death of Dr. H. B. Noble

**Published:** 1902-04

**Authors:** 


### DEATH OF DR. H. B. NOBLE.

THE announcement of the sudden death of Dr. Noble upon a street-car in Washington, D. C., on March 5, will carry with it a feeling of deep regret in dental circles throughout the country.

Dr. Noble was well known in the American Dental Association, as well as the present National. His kindly disposition and marked ability in the work of the various legislative positions he was called upon to fill in local, State, and national bodies endeared him to a large and ever-widening circle. In the city of his greatest activity—Washington, D. C.—he will be missed more than elsewhere, for there his power for good was more deeply felt.

The death of Dr. Noble leaves another vacant place in the ever-diminishing circle of the active men of the nineteenth century, and one more of those who made a part of the writer's professional life, has gone to his great reward for duties cheerfully and earnestly performed.

